# The missing-VP effect in readers of English as a second language

**DOI:** 10.3758/s13421-021-01159-0

**Published:** 2021-04-16

**Authors:** Stefan L. Frank, Patty Ernst, Robin L. Thompson, Rein Cozijn

**Affiliations:** 1 Centre for Language Studies, Radboud University, Nijmegen, The Netherlands; 2 School of Psychology, University of Birmingham, Birmingham, UK; 3 Tilburg Center for Cognition and Communication, Tilburg University, Tilburg, The Netherlands

**Keywords:** Sentence processing, Eye movements, Relative clauses, Second language reading, Grammaticality

## Abstract

English sentences with double center-embedded clauses are read faster when they are made ungrammatical by removing one of the required verb phrases. This phenomenon is known as the missing-VP effect. German and Dutch speakers do not experience the missing-VP effect when reading their native language, but they do when reading English as a second language (L2). We investigate whether the missing-VP effect when reading L2 English occurs in native Dutch speakers because their knowledge of English is similar to that of native English speakers (the *high exposure account*), or because of the difficulty of L2 reading (the *low proficiency account*). In an eye-tracking study, we compare the size of the missing-VP effect between native Dutch and native English participants, and across native Dutch participants with varying L2 English proficiency and exposure. Results provide evidence for both accounts, suggesting that both native-like knowledge of English and L2 reading difficulty play a role.

## Introduction

The missing-VP effect is the phenomenon that English double-embedded relative clauses, such as (1a), are not considered more acceptable and comprehensible than sentences such as (1b) that are ungrammatical because the second of three required verb phrases (VPs) has been deleted (Christiansen & MacDonald, 2009; Frank & Ernst, 2019; Gibson & Thomas, 1999). This example of a ‘grammaticality illusion’ (Phillips et al., 2011) has also been observed in French (Gimenes et al., 2009).

- The book that the student who the new catalog had confused a great deal was studying in the library was missing an important page.
*The book that the student who the new catalog had confused a great deal was missing an important page.

Gibson and Thomas (1999) argued that the missing-VP effect is caused by *structural forgetting*: After encountering three consecutive noun phrases (NPs), the comprehension system should predict that three VPs are still upcoming, but it is unable to keep all three predictions in working memory. One of the predictions is dropped and, consequently, only two VPs are expected. According to Gibson and Thomas (1999), the prediction from the second NP is dropped because this frees up most memory. This explains why the missing-VP effect only occurs when it is the second VP that is missing (Frank & Ernst, 2019; Gibson & Thomas, 1999). An alternative explanation for the special role of the second VP is provided by Häussler and Bader (2015), who related it to well-established primacy and recency effects in working memory: The first and last items of a list are remembered better than intermediate items, so the middle NP’s verb prediction is most likely to be forgotten. This explanation once again highlights the role of working memory limitations in causing the missing-VP effect.

The missing-VP effect is not only observed in subjective judgements about sentences but also in reading times. In a series of eye-tracking and self-paced reading studies, Vasishth et al., (2010) found longer reading times on the final verb (and beyond) in grammatical compared to ungrammatical double-embedded English sentences. However, when testing native German speakers in German equivalents of the sentences, the effect was reversed: They showed increased reading times for *ungrammatical* sentences. Vasishth et al., (2010) explained this absence of the grammaticality illusion in German by the fact that relative clauses are always verb-final in that language, and indeed the finding was later replicated in Dutch, another language with verb-final relative clauses (Frank et al.,, 2016; see also Frank and Ernst (2019), for evidence from sentence rating tasks). Possibly, German and Dutch speakers are so used to keeping verb predictions in working memory that they are less prone to structural forgetting than English speakers.

If this is indeed the case, we would expect German and Dutch speakers to be able to also keep verb predictions in memory when processing double-embedded relative clauses in English as a second language (L2), and, therefore, not show the missing-VP effect in English either. However, the opposite has been found: When L1 German or Dutch speakers are tested in L2 English, they do show the effect like native English speakers do (Frank et al., 2016, 2019). This finding led Frank et al., (2016) to argue that the difference between the reading-time patterns in English and German/Dutch is not in fact caused by English speakers being more prone to structural forgetting than German/Dutch speakers but by aspects of the statistical patterns of these languages, such as the much higher probability of encountering three consecutive verb phrases in German or Dutch than in English. That is, whether or not the missing-VP effect occurs directly depends on properties of the *language* rather than properties of the *participants* (i.e., their L1-dependent propensity for structural forgetting). Both native and non-native English readers form expectations based on the learned language statistics, so sufficient exposure to English will make the non-natives pattern like native readers. That is, exposure to a large enough sample of English sentences leads to the missing-VP effect, both in L1 and L2 readers. In the current paper, we call this the *high exposure account*. It predicts that higher exposure to English will increase the size of the missing-VP effect, irrespective of the participant’s native language, and that L1 participants show a larger effect than L2 English participants, all other things being equal.

The possibility remains, however, that the L1 German and L1 Dutch speakers of the Frank et al., (2016) study only showed the missing-VP effect in L2 English because of their relatively low English proficiency. According to this *low proficiency account*, native speakers of German and Dutch are less sensitive to structural forgetting than native English speakers, but nevertheless show the missing-VP effect in L2 English because sentence processing is more taxing for working memory in L2 than L1. This is known to be the case in particular for participants with lower L2 proficiency (Hopp, 2014; Service et al., 2002). Hence, the L2 English speakers would *not* have suffered from structural forgetting if they had been as proficient in English as in their L1. This account predicts that L2 English participants with higher English proficiency are less sensitive to the grammaticality illusion, or may not even experience the illusion at all. Under this account, the overall difference between native and non-native participants depends on how strongly the two groups differ in their English proficiency and their sensitivity to structural forgetting. If the non-natives are much less proficient but only a little less prone to structural forgetting than the native English readers, the low-proficiency account predicts the missing-VP effect to be larger in L2 than in L1 English. Conversely, if the L2 English proficiency of L1 Dutch/German speakers is close to that of native English speakers but they are much less likely to forget a VP prediction, the missing-VP effect would be smaller in L2 English than in L1 English. Hence, we cannot derive from the low proficiency account a prediction about the difference in the missing-VP effect between the two groups.

Frank et al., (2016) reject the low-proficiency account for being less parsimonious because it seems to imply that different causes underlie the missing-VP effect in native and non-native readers. Moreover, their data indicated a smaller missing-VP effect for their L1 German than the L1 Dutch participants, even though the latter scored significantly higher on an English proficiency test and (unlike the L1 German participants) they were all students of English Language and Culture.

The current study directly tests the two accounts of the missing-VP effect in L2 English. Native English- and native Dutch-speaking participants took part in an eye-tracking study in which they read grammatical English sentences with double-embedded relative clauses, as well as their ungrammatical (missing-VP) counterparts. We expected the missing-VP effect to be stronger in the L1 English than L1 Dutch group, as predicted by the high-exposure account, but found only sporadic evidence for this. Next, we analyzed the effects of L2 English proficiency and exposure (as measured by a reading test and language background questionnaire) within the L1 Dutch group, expecting to find that the size of the missing-VP effect increases with higher exposure but does not depend on proficiency (after correcting for exposure differences). However, we found that higher proficiency does lead to a stronger missing-VP effect, although there was also weak evidence for stronger missing-VP effect with higher exposure. These results more strongly support the low-proficiency account than the high-exposure account, and suggest that both working memory and language statistics need to be taken into account for explanations of the missing-VP effect.

## Method

### Materials

#### Target sentence structures

We constructed 24 grammatically correct English target sentences with double-embedded object-relative clauses. Twelve of these (the semantically “neutral” sentences) had nouns and verbs that allowed for many meaningful combinations of agent, action, and patient. The nouns and verbs of the other 12 sentences (semantically “biased”) were such that most (if not all) unintended combinations are not meaningful. For each sentence, an ungrammatical version was created by removing the second verb phrase. Hence, there were 2 (Grammaticality) × 2 (Semantics) = 4 item conditions. Table 1 presents one example in each condition. The full list of target items is presented in the Appendix.^1^

**Table 1:** Stimulus sentence example in each item condition (N = Neutral, B = Biased, G = Grammatical, U = Ungrammatical)

Sem.	Gramm.	Sentence
N	G	The carpenter who the craftsman who the peasant carried a long way hurt on
		purpose supervised the apprentice in the garden.
N	U	The carpenter who the craftsman who the peasant carried a long
		way supervised the apprentice in the garden.
B	G	The book that the student who the new catalog had confused a great deal was studying
		in the library was missing an important page.
B	U	The book that the student who the new catalog had confused
		a great deal was missing an important page.

Line breaks are as presented in the experiment. Underlined phrases are the two regions of interest (underlining was not visible to participants)

The semantically biased and neutral sentences were based on the stimuli from Gibson and Thomas (1999) and Frank et al., (2016), respectively. Some of Gibson and Thomas’s items contained words that we suspected to be unknown to many of our non-native participants. These words (e.g., ‘snubbed’) were replaced by better known alternatives. Adverbs were added to the Frank et al., sentences to make them more similar to the semantically biased ones.

We had no reason to expect an interaction between Grammaticality and Semantics. The missing-VP effect has been demonstrated in both semantically neutral (Christiansen & MacDonald, 2009; Frank et al., 2016; Vasishth et al., 2010) and semantically biased sentences (Christiansen & MacDonald, 2009; Frank & Ernst, 2019; Gibson & Thomas, 1999), although reading-time studies have been restricted to neutral sentences (at least in English). The shallow structure hypothesis (Clahsen & Felser, 2006a, b) claims that non-native readers rely more on semantic cues when syntactic structure is highly complex. If so, we might expect our L1 Dutch participants to show a weaker missing-VP effect (or even no such grammaticality illusion) on the semantically biased sentences.

#### Regions of interest

We defined two regions of interest (RoI), as shown in the Appendix for each target stimulus. Region **V3** comprises the final verb or auxiliary-verb pair (e.g., ‘was missing’ in Table 1) and region **post-V** are all words following V3 (e.g., ‘an important page.’ in Table 1). Vasishth et al., (2010) found a missing-VP effect on re-reading times in both these regions.

For consistency with Vasishth et al., (2010), we had originally also included a V1 region consisting of the first verb or auxiliary-verb pair, even though grammatical and ungrammatical sentences are the same up to and including this RoI so first-pass effect of Grammaticality are not expected here. However, the length of the text line following V1 confounds with Grammaticality (see Table 1 and the upcoming paragraph), which may lead to an illusory effect of Grammaticality. Because we indeed found that the length of the remaining text line affects V1 reading time, we do not report results on the V1 region here. They are available as supplementary materials from https://osf.io/ye6dj.

#### Line breaks

Target sentences did not comfortably fit on a single screen line, so line breaks had to be inserted. To make sure that the screen position of the critical V3 and post-V regions was approximately the same for grammatical and ungrammatical items, the location of the line break differed between Grammaticality conditions: within the middle verb phrase for grammatical sentences but shortly (or immediately) after the first main verb for ungrammatical items. There was at least one content word to the left of the V3 region, discouraging the return sweep from landing inside the V3 region. Long words or phrases in the original stimuli (Frank et al., 2016; Gibson and Thomas, 1999) were shortened if required to make the first line fit on screen (e.g., ‘ancient manuscript’ was replaced by ‘book’).

As can be seen in Table 1, the spatial location of the critical regions was not always identical between Grammaticality conditions. On average, they were positioned 1.8 characters (*SD* = 3.1) more to the right in the grammatical condition. In principle, this could have an influence on the main effect of Grammaticality. However, there is no reason to expect that it will affect the critical interactions with native language, L2 English proficiency, and L2 English exposure.

#### Lists

We constructed a first stimulus list with 24 target items (six from each condition) that were evenly distributed among 96 filler sentences. A second list was identical to the first but with the opposite Grammaticality conditions. Two further lists were created by reversing the order of the first two lists. Participants were assigned randomly to one of the four lists.

Thirty sentences (six targets and 24 fillers) were paired with a yes/no comprehension question intended to ensure participants read attentively. No two consecutive items appeared with a comprehension question.

### Participants

The experiment was completed by 197 participants. Following the pre-registered analysis, 14 of these were excluded because they scored below 70% correct on the comprehension questions; one additional participant was excluded because of persistent calibration failure. Of the remaining participants, 58 were native English speakers (L1 English group: 41 females, 11 males, six other/unknown; mean age 20.2, range 18–26). The other 124 were native Dutch speakers with English as a second language (L1 Dutch group: 84 females, 40 males; mean age 22.3, range 18–57). To obtain a wide range of L2 English exposures for this rather homogeneous group, we explicitly recruited participants studying in bachelor or master programs that are (nearly) exclusively taught in Dutch or fully taught in English.

All participants filled out a language background questionnaire and completed the Vernon-Warden reading test (VWRT; Hedderly, 1996) for English reading proficiency, which is a timed test of increasingly challenging fill-in-the-blanks multiple choice questions.

#### Group comparison

**Fig. 1:**
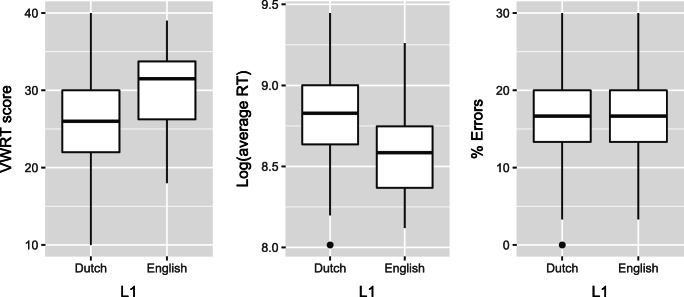
Boxplots of Vernon-Warden reading test scores (*left*), average log-transformed reading time on filler sentences (*middle*), and error rate (*right*), for each L1 group

Figure 1 shows how the two groups performed on three dimensions of English proficiency: VWRT score, reading speed (operationalized as average total reading time on filler sentences), and comprehension accuracy (percentage of errors on comprehension questions). The L1 English group read faster and scored higher on the VWRT, although both measures show considerable overlap between groups. There is no difference between groups whatsoever in comprehension accuracy, and error rates were similar to those reported by Vasishth et al., (2010) (L1 English participants) and Frank et al., (2016) (L1 Dutch participants tested in L2 English).

### L2 English proficiency and exposure

The L1 Dutch participants’ VWRT results and language background questionnaires were used to obtain scores of their L2 English proficiency and exposure/use. The overall proficiency score was computed by running a principal component analysis (PCA) over z-scores of five separate L2 English proficiency measures:^2^ VWRT scores and self-rated (seven-point scales) English proficiency in speaking, listening, reading, and writing.

PCA is a well-known method for summarizing high-dimensional data in a lower number of dimensions, called PCA *components*. These components are ordered by how much unique variance in the original data they explain: The first PCA component explains the largest amount of variance. Hence, we took this first component to comprise the participants’ overall Proficiency scores; it explained 62% of variance in the five proficiency measures. As an additional output of PCA, each of the original measures receives a so-called *factor loading* indicating to what extent the measure contributes to the different PCA components. For the first PCA component that we use as a proficiency measure, the factor loadings on the four self-rated proficiencies were approximately equal (0.43 − 0.50) and slightly higher than the factor loading on VWRT scores (0.35).

Similarly, an overall L2 English exposure measure was computed by running a PCA on the questionnaire data related to amount of exposure to (and use of) English as a second language: self-rated (seven-point scales) amount of English used for speaking, listening, reading, and writing; number of years since first exposure to English and since first formal schooling in English; estimated hours per week English use in classes, reading for study, reading for leisure, and listening/speaking; estimated percentage of reading in English for study and for leisure; and number of English-taught classes in the current study year.^3^ The first PCA component comprised the participants’ overall Exposure scores; it explained 46% of variance in the 13 exposure measures. Factor loadings were between 0.22 − 0.34 for all measures except for number of years since first English exposure and schooling, which both had slightly negative loadings.

#### Validating the proficiency and exposure measures

**Fig. 2:**
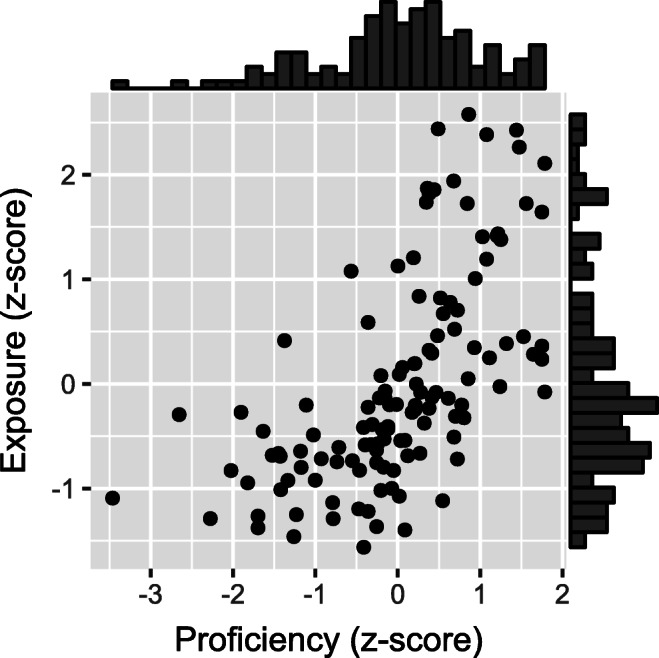
Scatter plot of Proficiency against Exposure scores, with histograms for both scores, for participants included in the analysis

As expected, Proficiency and Exposure were positively correlated (*r* = .61 over all L1 Dutch participants; *r* = .62 over participants included in the analysis; see Fig. 2).

If the Proficiency score is indeed a valid measure of true L2 English proficiency, it should correlate positively with reading speed and/or comprehension accuracy. Whether this is the case of the Exposure score is less clear because any positive effect of increased L2 English exposure may already be incorporated in the Proficiency score. To validate the two scores, we looked at the correlations between each of the Proficiency and Exposure scores on the one hand, and the two behavioural measures (log-transformed average filler sentence RT and percentage of errors on comprehension questions) on the other. As shown in Table 2, higher Proficiency leads to fewer errors and higher Exposure leads to shorter reading times. However, the partial correlations, where Exposure is partialled out from Proficiency or vice versa, show that the relation between Exposure and reading time does not survive the correction for Proficiency. We will return to the issue of Proficiency/Exposure validity in the Discussion.

**Table 2:** 95% Confidence intervals of correlation coefficients (Pearson’s *r*) between Proficiency/Exposure scores and two behavioural measures, for participants included in the analysis

	Reading time		% Error	
	Simple	Partial	Simple	Partial
Proficiency	[−.32, +.03]	[−.23, +.13]	[−.48,−.16]	[−.47,−.12]
Exposure	[−.34,−.00]	[−.29, +.07]	[−.33, +.02]	[−.12, +.24]

### Procedure

All L1 Dutch speakers and eight of the L1 English speakers were tested at the Centre for Language Studies lab of Radboud University, Nijmegen. They received €10 or course credit for their participation. All other L1 English-speaking participants were tested at the Multimodal Multilingual Language Processing lab at the University of Birmingham. They received *£*7 for their participation.

Participants were seated with their head in a chin rest, at a distance of 50 cm from the SR Research EyeLink 1000+ eye tracker. An instruction screen then informed the participants they would read 120 sentences, one at a time, with a break halfway. Participants were instructed to look at the fixation point until the sentence appeared and to read it in a natural fashion. After reading the sentence, they had to press the space bar and answer the yes/no question (if any) by means of a key press. After successful nine-point calibration, five practice sentences with two practice questions followed. After 60 trials, the participants were given the opportunity to have a break. After the break, another calibration was performed, and the participants proceeded with reading the remaining 60 sentences.

Each trial consisted of a fixation point on the left side of the screen, where the first word of the sentence would appear. This fixation point was simultaneously a correction for small drifts in the gaze position. The sentences appeared when the gaze approached the fixation point close enough for the experimenter to accept the drift correction. The participants pressed the space bar when they had read and understood the sentence. Stimuli were presented in 18-point Calibri font. If a sentence was followed by a question, the word ‘question’ was presented with the question underneath, and below that the words ‘yes’ and ‘no’ with their corresponding response keys (‘z’ and ‘m’, respectively).

The eye tracking took approximately 30 minutes, including set up and calibration. Following this phase of the experiment, participants filled out the background questionnaire and completed the VWRT. A complete session could take up to 1 hour. The study was approved by the Ethics Assessment Committee Humanities of Radboud University.

### Data analysis

#### Preprocessing

The EyeLink tracker software automatically assigns fixations to words. However, because of drifts or imperfect calibration, fixations can systematically land too far above or below the text to be assigned to a word. For this reason, all fixations were checked by a research assistant. Using the software Fixation (Cozijn, 2006), these unassigned fixations were moved vertically to assign them to words. Such adjustments were rarely required: only 0.12% and 0.05% of fixations were reassigned for the L1 Dutch and L1 English participants, respectively. Trials were marked as not usable if it could not reliably be determined which fixations belonged to which words. These trials (2.55% for L1 Dutch, 1.15% for L1 English) were excluded from analysis.

#### Reading time measures

We analyzed effects on one early and one later reading time (RT) measure: first-pass RT and regression-path RT. The regression-path RT for a RoI is defined as the time between the first fixation in the RoI and the first fixation on a word to the right of the RoI. Both first-pass and regression-path RT are 0 if (and only if) the RoI is skipped in first pass. These data were excluded from analysis. Non-zero RTs were only excluded from analysis if they were extremely long: Over 4 s for first-pass RT and over 20 s for regression-path RT. Regression paths may include multiple re-readings over long stretches of the sentence, leading to very long, but not unrealistic, RTs. The percentages of data points that were excluded because of extreme RTs can be found in Table 3.

**Table 3:** Percentage of data points excluded because RT is 0 or above threshold

		Region of Interest	
Measure	RT	V3	post-V
First pass	0 ms	7.88%	0.69%
	> 4 s	0%	0.09%
Regression path	0 ms	5.94%	0.69%
	> 20 s	0%	1.03%

The ‘above threshold’ percentages are computed after removing the 0 ms data

#### Regression models

For each RT measure and each RoI, two Bayesian mixed-effects regression models were fitted using the R package brms (v.2.8.0, Bürkner, 2017). The first model compared the L1 Dutch and L1 English participant groups, the second analyzed effects of Proficiency and Exposure within the L1 Dutch group.

Between-group analyses included factors for Grammaticality, Semantics, and L1, plus all two-way interactions and the three-way interaction between them. Subject and item were included as random effects, with by-subject random slopes of Grammaticality and Semantics and by-item random slopes of Grammaticality and L1. For the L1 Dutch group analysis, we included Grammaticality, Semantics, Proficiency, and Exposure as predictors. Two- and three-way interactions were included if they did not contain both Proficiency and Exposure. Further, there were by-subject random slopes of Grammaticality and Semantics and by-item random slopes of Grammaticality, Proficiency, and Exposure.

Proficiency and Exposure scores were standardized (z-scores). The factor levels for Grammaticality, Semantics, and L1 were coded as − 0.5 (Ungrammatical, Neutral, Dutch) and + 0.5 (Grammatical, Biased, English). This means that a non-negative coefficient of Grammaticality is indicative of the missing-VP effect (no shorter RTs in grammatical than ungrammatical sentences) and a positive interaction between Grammaticality and L1 means that the effect is stronger for the L1 English group, as predicted by the high-exposure account. The high-exposure account further predicts a positive interaction between Grammaticality and Exposure, while the low-proficiency account predicts a *negative* interaction between Grammaticality and Proficiency.

Because RTs are always positive and their distribution right-skewed, we should not assume normally distributed data. Posterior predictive checks (see supplementary materials) revealed that a Gamma distribution was most appropriate for the post-V region while a shifted log-normal distribution worked best for the V3 region, so these two distributions were assumed for the respective regions.^4^

The number of iterations for model fitting was set to 3000 (of which 1000 are warmup) and control parameters were set to the brms defaults. Priors, too, were the brms defaults except for the intercept priors. which were normally distributed with means of 6 (on a log-ms scale) for first-pass RT on the V3 region, 7 for regression-path RT on V3 and first-pass RT on post-V, and 8 for regression-path RT on post-V. All intercept priors had a standard deviations of 1.

#### Deviations from preregistration

As listed below, our analysis deviates in several respects from what was preregistered (see https://osf.io/ye6dj). Results from the preregistered analysis are presented in the supplementary materials. These yield the same conclusions as the current analysis.

- After preregistration, we had the opportunity to test 20 additional L1 Dutch participants. Considering we aim to detect (possibly very small) effects of individual differences between L1 Dutch participants, we chose to include the additional participants. Bayesian data analysis (unlike traditional null-hypothesis testing) allows for incrementally increasing the amount of data.
- According to the preregistration, only re-reading time (equal to total RT minus first-pass RT) would be used as a later RT measure. Re-reading times of 0 occur when a RoI is not fixated on after first pass. However, it turned out that over 50% of re-reading times would be excluded for this reason. This motivated us to use regression-path RT instead, which yields much less data loss. Liversedge et al., (1998) recommend both re-reading time and regression-path RT as measures of recovery from reading difficulty.
- The preregistration claims that all priors were the brms defaults, but this was not the case for the intercepts.
- Gamma distributions (as recommended by Lo and Andrews, 2015) were preregistered but one anonymous reviewer noted it is more common to assume a log-normal distribution for reading times. As described above, we settled on the shifted log-normal distribution for the V3 region.

## Results

### Between-group comparison

**Fig. 3:**
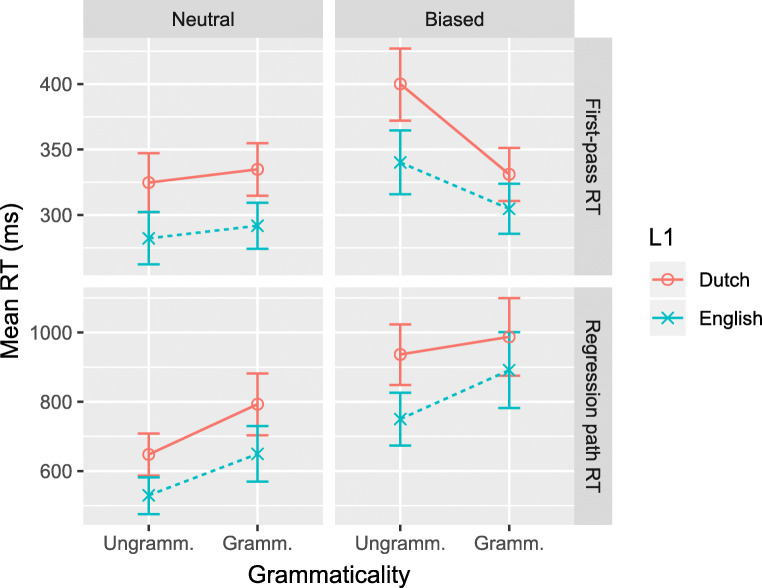
Fitted mean reading times with 68% credible interval for between-group comparison on V3 region

**Fig. 4:**
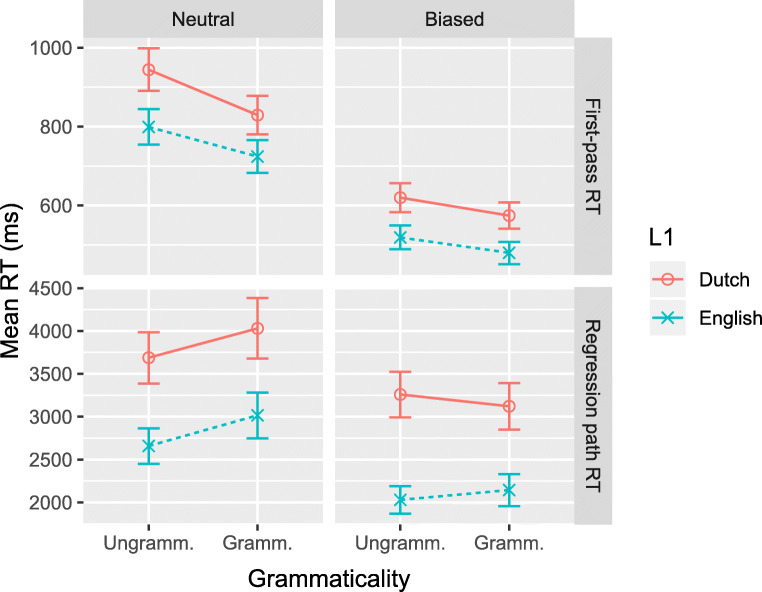
Fitted mean reading times (in ms) with 68% credible interval for between-group comparison on post-V region

Figures 3 and 4 show the results of comparisons between the native Dutch and native English-speaking participants, on the V3 and post-V regions, respectively. Each plot displays the fitted mean RTs for the two sentence types and for both reading measures. Regression analysis results are presented in Tables 4 and 5, which show the fitted coefficient, its 95% credible interval, and the posterior probability that the coefficient is positive (i.e., *P*(*b* > 0); as recommended by Makowski et al., (2019), and Nicenboim and Vasishth (2016)).^5^ The full posterior probability distributions are presented in the supplementary materials.

**Table 4:** Regression model fixed effects for between-group comparison at V3 region

	First-pass RT			Regression path RT		
	*b*	95% CrI	*P*(*b* > 0)	*b*	95% CrI	*P*(*b* > 0)
Grammaticality	− 0.051	[− 0.136, 0.033]	.11	0.180	[ 0.038, 0.326]	.99
Semantics	0.112	[− 0.042, 0.260]	.93	0.345	[ 0.061, 0.627]	.99
L1	− 0.143	[− 0.224,− 0.060]	.00	− 0.210	[− 0.326,− 0.095]	.00
Gram.×Sem.	− 0.170	[− 0.336,− 0.005]	.02	− 0.077	[− 0.352, 0.200]	.29
Gram.×L1	0.044	[− 0.048, 0.135]	.83	0.071	[− 0.058, 0.200]	.86
Sem.×L1	0.018	[− 0.076, 0.113]	.65	0.048	[− 0.104, 0.205]	.73
Gram.×Sem.×L1	0.083	[− 0.084, 0.247]	.83	0.132	[− 0.076, 0.335]	.90

**Table 5:** Regression model fixed effects for between-group comparison on post-V region

	First-pass RT			Regression path RT		
	*b*	95% CrI	*P*(*b* > 0)	*b*	95% CrI	*P*(*b* > 0)
Grammaticality	− 0.093	[− 0.143,− 0.044]	.00	0.069	[− 0.002, 0.140]	.97
Semantics	− 0.413	[− 0.529,− 0.291]	.00	− 0.270	[− 0.390,− 0.156]	.00
L1	− 0.176	[− 0.279,− 0.069]	.00	− 0.392	[− 0.593,− 0.180]	.00
Gram.×Sem.	0.031	[− 0.066, 0.127]	.74	− 0.090	[− 0.212, 0.033]	.08
Gram.×L1	0.016	[− 0.063, 0.094]	.66	0.072	[− 0.039, 0.183]	.90
Sem.×L1	− 0.030	[− 0.115, 0.055]	.25	− 0.124	[− 0.250, 0.003]	.03
Gram.×Sem.×L1	− 0.036	[− 0.191, 0.121]	.33	0.065	[− 0.124, 0.255]	.74

#### Region V3

Consistent with Vasishth et al., (2010), the missing-VP effect is not visible in first-pass RTs of semantically neutral sentences. In contrast, regression-path RT shows strong evidence of the missing-VP effect: Reading slows down when the sentence is grammatical. There is no evidence for an interaction with Semantics or L1, and at best very weak evidence for the three-way interaction.

#### Region post-V

The post-V region is read faster in first pass when the sentence is grammatical than when it is not, that is, there is no illusion of grammaticality. On the later reading-time measure, there is evidence for a missing-VP effect but only for semantically neutral sentences. This was confirmed by separate analyses for the Neutral and Biased sentences (Neutral: *b*_Gram_ = 0.11;CrI = [0.03,0.19];*P*(*b* > 0) = .998. Biased: *b*_Gram_ = 0.02;CrI = [− 0.12,0.15];*P*(*b* > 0) = .61). There are no differences of interest between the two participant groups.

### L1 Dutch group analysis

To assess whether the collinearity between the Proficiency and Exposure predictors could cause problems with fitting or interpreting the regression models, we computed Variance Inflation Factors (VIF) using the function vif.mer.^6^ For both the regions of interest and both the RT measures, all VIF were below 2, indicating there was no serious issue of collinearity (Sheather (2009), regards VIF above 5 as potentially problematic).

**Fig. 5:**
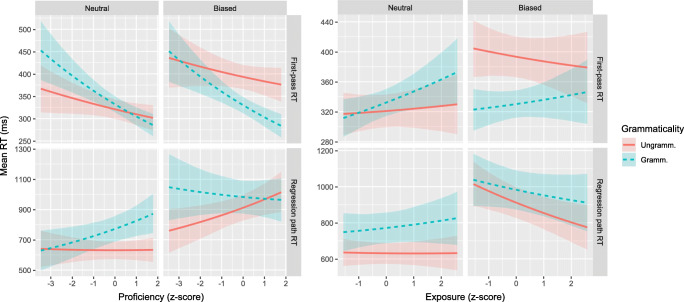
Fitted mean reading times with 68% credible interval for L1 Dutch group on V3 region, as a function of Proficiency (*left*) or Exposure (*right*)

**Fig. 6:**
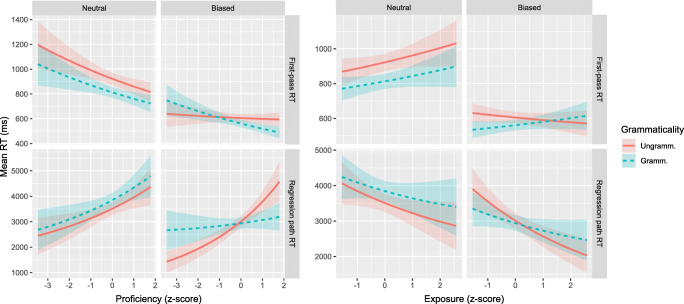
Fitted mean reading times with 68% credible interval for L1 Dutch group on post-V region, as a function of Proficiency (*left*) or Exposure (*right*)

Figures 5 and 6 show the results of the analyses of Proficiency and Exposure effects in the L1 Dutch participants, on the V3 and post-V regions of interest, respectively. Each plot displays the fitted mean RTs for all Grammaticality and Semantics conditions as a function of Proficiency or Exposure. Regression analysis results are presented in Tables 6 and 7, which show the fitted coefficient, 95% credible interval, and the posterior probability that the coefficient is positive. The full posterior probability distributions are available as supplementary materials.

**Table 6:** Regression model fixed effects for L1 Dutch group on V3 region

	First-pass RT			Regression path RT		
	*b*	95% CrI	*P*(*b* > 0)	*b*	95% CrI	*P*(*b* > 0)
Grammaticality	− 0.070	[− 0.158, 0.016]	.05	0.146	[− 0.004, 0.292]	.97
Semantics	0.100	[− 0.053, 0.250]	.90	0.325	[ 0.057, 0.596]	.99
Proficiency	− 0.060	[− 0.115,− 0.003]	.02	0.029	[− 0.047, 0.107]	.77
Exposure	0.013	[− 0.045, 0.069]	.68	− 0.021	[− 0.097, 0.054]	.29
Gram.×Sem.	− 0.212	[− 0.384,− 0.034]	.01	− 0.138	[− 0.419, 0.150]	.16
Gram.×Prof.	− 0.056	[− 0.120, 0.007]	.04	− 0.002	[− 0.095, 0.089]	.48
Gram.×Expo.	0.034	[− 0.034, 0.097]	.84	0.031	[− 0.064, 0.124]	.75
Sem.×Prof.	0.005	[− 0.063, 0.073]	.55	− 0.012	[− 0.104, 0.081]	.40
Sem.×Expo.	− 0.026	[− 0.096, 0.043]	.23	− 0.063	[− 0.158, 0.034]	.09
Gram.×Sem.×Prof.	− 0.010	[− 0.134, 0.117]	.44	− 0.144	[− 0.291, 0.004]	.03
Gram.×Sem.×Expo.	− 0.001	[− 0.127, 0.127]	.49	0.009	[− 0.143, 0.157]	.55

**Table 7:** Regression model fixed effects for L1 Dutch group on post-V region

	First-pass RT			Regression path RT		
	*b*	95% CrI	*P*(*b* > 0)	*b*	95% CrI	*P*(*b* > 0)
Grammaticality	− 0.101	[− 0.151,− 0.051]	.00	0.034	[− 0.034, 0.102]	.84
Semantics	− 0.397	[− 0.522,− 0.275]	.00	− 0.212	[− 0.333,− 0.089]	.00
Proficiency	− 0.058	[− 0.131, 0.015]	.06	0.125	[− 0.021, 0.273]	.95
Exposure	0.021	[− 0.054, 0.096]	.71	− 0.097	[− 0.249, 0.052]	.11
Gram.×Sem.	0.051	[− 0.049, 0.153]	.85	− 0.115	[− 0.243, 0.010]	.03
Gram.×Prof.	− 0.031	[− 0.089, 0.029]	.15	− 0.093	[− 0.169,− 0.016]	.01
Gram.×Expo.	0.026	[− 0.034, 0.087]	.81	0.057	[− 0.022, 0.138]	.92
Sem.×Prof.	0.024	[− 0.038, 0.086]	.78	0.017	[− 0.098, 0.133]	.62
Sem.×Expo.	− 0.035	[− 0.097, 0.028]	.13	− 0.047	[− 0.158, 0.067]	.19
Gram.×Sem.×Prof.	− 0.071	[− 0.188, 0.046]	.11	− 0.188	[− 0.328,− 0.043]	.00
Gram.×Sem.×Expo.	0.063	[− 0.055, 0.181]	.86	0.053	[− 0.095, 0.197]	.77

#### Region V3

There is weak evidence for a negative interaction between Grammaticality and Proficiency on first-pass RT, suggesting that a missing-VP effect (if any) might be stronger in low-proficient than in high-proficient readers. The missing-VP effect on first-pass RT does not reliably vary with L2 English exposure.

The possible three-way interaction with Proficiency in regression-path RTs suggests that lower Proficiency increases the missing-VP effect for semantically biased sentences but decreases it for the neutral sentences (see the two bottom left panels of Fig. 5). However, separate analyses for the Biased and Neutral sentences revealed at most very weak evidence for interactions between Grammaticality and Proficiency (Biased: *b*_Gram×Prof_ = − 0.07;CrI = [− 0.17,0.03];*P*(*b* > 0) = .09. Neutral: *b*_Gram×Prof_ = 0.07;CrI = [− 0.07,0.21];*P*(*b* > 0) = .84).

#### Region post-V

There is a negative interaction between Grammaticality and Proficiency on regression path RTs: The missing-VP effect is stronger for less proficient readers, albeit only for semantically biased sentences (Biased sentences only: *b*_Gram×Prof_ = − 0.19;CrI = [− 0.32,− 0.06];*P*(*b* > 0) = .002. Neutral only: *b*_Gram×Prof_ = 0.01;CrI = [− 0.10,0.11];*P*(*b* > 0) = .53). There is very weak evidence for a positive interaction between Grammaticality and Exposure on regression path RTs, suggesting a possibly stronger missing-VP effect with higher exposure.

## Discussion

We replicated Vasishth et al.,’s (2010) finding that the third verb (V3 region) of a grammatical double-embedded sentence is read more slowly than the same region of a sentence that is ungrammatical because the second verb phrase has been removed. This missing-VP effect is apparent only on the later reading-time measure extracted from the eye-tracking signal, again mirroring the results by Vasishth et al., (2010). In an eye-tracking study comparing single-embedded relative clauses to non-embedded subject-relatives, Gordon et al., (2006), too, found that comprehension difficulty on the matrix verb was apparent in regression-path RTs but not first-pass RTs. Hence, earlier studies found evidence that comprehension difficulty caused by embedded clauses shows up only in later RT measures, and our results are consistent with this evidence.

The missing-VP effect also appeared on the post-verbal region of semantically neutral sentences, possibly because of spillover from the V3 region. The absence of spillover on semantically biased sentences might be due to their longer V3 regions compared to those of the semantically neutral sentences: Spillover from the V3 region of semantically neutral sentences necessarily leads to effects in the post-V region, whereas in semantically biased sentences spillover may move effects to a later part of V3, rather than showing up in post-V.

### Between-group comparison

Like Frank et al., (2016), we found that L1 Dutch participants display the missing-VP effect in L2 English. The high-exposure account of the missing-VP effect in L2 English predicts that the effect is stronger in native than non-native English readers. Our comparison between L1 Dutch and L1 English participant groups did not reveal evidence for this. Hence, the between-group comparison failed to consistently support the high-exposure account.

### L1 Dutch group analysis

The high-exposure account predicts a stronger missing-VP effect as L2 English exposure increases. The low-proficiency account predicts a *weaker* missing-VP effect as L2 English proficiency increases. Our results do not strongly support either account although they do provide some (albeit dispersed and often weak) evidence for both.

First-pass RTs on the V3 region do not reveal a missing-VP effect in general but the possible interaction between Grammaticality and Proficiency (see Figure 5, two top left panels) suggests that the effect occurs in low-proficiency readers, as predicted by the low-proficiency account. The post-V region shows the strongest evidence for the low-proficiency account: The missing-VP effect on regression-path RTs decreases with higher proficiency; an effect that is driven by the semantically biased sentences.

The only evidence (albeit very weak) for the high exposure account comes from regression-path RTs in the post-V region, where the missing-VP effect appears to increase with higher exposure to L2 English.

To summarize, we found sporadic evidence in support of the low-proficiency account and no evidence opposing it. Likewise, at no point do we find that the missing-VP effect decreases with higher exposure, which would constitute evidence against the high-exposure account. However, in light of the weakness of the evidence as well as the multiple comparison issue caused by analyzing two RT measures on two sentence regions (Von der Malsburg and Angele, 2017), one should exercise caution when drawing conclusions from these results.

### Combining the proficiency and exposure measures

Although our evidence for the low-proficiency and high- exposure accounts is very limited, it is noteworthy that, to the extent that Proficiency and Exposure interact with Grammaticality, the directions of these interactions go in opposite directions—the directions predicted by the two accounts (see Tables 6 and 7). This raises the question whether both accounts may be correct, that is, L1 Dutch readers display a stronger missing-VP effect in L2 English if they have lower proficiency but also if they have higher exposure. If so, this will be difficult to discover from individual differences in proficiency and exposure because these show a fairly strong positive correlation (*r* = .62 for our measures).

The Proficiency and Exposure scores were validated by their correlations with error rate and overall reading time, respectively (see Table 2). However, partialling out Proficiency weakened the correlation between Exposure and reading times, which suggests that the Exposure score does not make a substantial contribution to what Proficiency already captures. If our Exposure score is indeed not much more than Proficiency plus noise, this would explain why its effects are (nearly) undetectable.

**Fig. 7:**
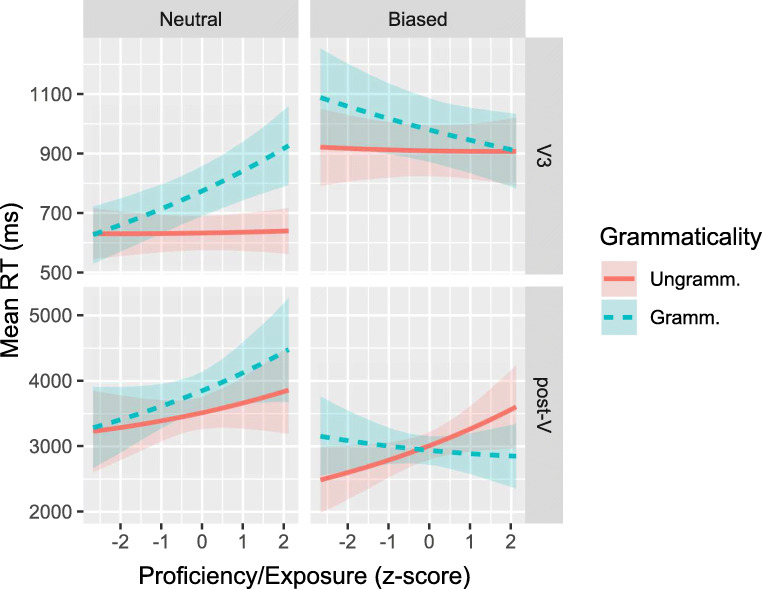
Fitted mean regression-path RTs with 68% credible interval for L1 Dutch group as a function of the combined Proficiency/Exposure score. *Top row*: V3 region; *bottom row*: post-V region

We thus ran post-hoc analyses in which we replaced the separate Proficiency and Exposure scores by a single measure, obtained by taking the first component from a PCA on the combination of VWRT scores and English proficiency and exposure/use questionnaire responses.^7^ If this combined score shows consistent, positive interactions with Grammaticality, this would constitute evidence for the high-exposure and against the low-proficiency account. Conversely, negative interactions would be evidence against the high-exposure and in support of the low-proficiency account. However, there is no interaction between Proficiency/Exposure and Grammaticality whatsoever in first-pass RTs. As can be seen in Fig. 7, on regression-path RTs the direction of interactions depends on the sentence type: the three-way interactions are reliable both on the V3 (*b* = − 0.12;CrI = [− 0.24,− 0.00];*P*(*b* > 0) = .02) and post-V (*b* = − 0.13;CrI = [− 0.24,− 0.01];*P*(*b* > 0) = .01) regions. Separate analyses on semantically neutral and biased sentences revealed (very) weak evidence for two-way interactions only for semantically neutral sentences in the V3 region (*b*_Gram×Prof/Expo_ = 0.08;CrI = [− 0.03,0.19];*P*(*b* > 0) = .92) and for biased sentences in the post-V region (*b*_Gram×Prof/Expo_ = − 0.10;CrI = [− 0.20,0.00];*P*(*b* > 0) = .028). The absence of reliable and consistent interactions between Grammaticality and Proficiency/Exposure could be symptomatic of proficiency and exposure effects going in opposite directions and cancelling each other out.

### Memory- and expectation-based accounts of the missing-VP effect

Taken together, the between-group comparison and analyses of L2 English proficiency and exposure effects revealed more evidence in support of *both* the high-exposure *and* the low-proficiency account than against either account. We may therefore tentatively conclude that there is truth in both explanations of the missing-VP effect in L2 English by L1 Dutch readers: The effect is caused by exposure to the structural patterns of English but also by the lower proficiency in L2 (compared to L1) leading to higher working-memory demands during reading (Hopp, 2014; Service et al., 2002). This implies that in native English readers, too, the missing-VP effect is driven by both language-statistics-based expectations and working-memory limitations, as was also suggested by Christiansen and Chater (2016), and Frank and Ernst (2019).

Further support for the roles of both linguistic expectations and memory limitations in generating the missing-VP effect comes from simulations with recurrent neural networks (RNNs) for sentence processing (Elman, 1990). When these models are trained on next-word prediction, they generate probabilistic, language-statistics-based expectations about the upcoming word at each point in the sentence. These models also suffer from ‘working memory’-limitation in that their next-word predictions are often not sensitive enough to much earlier parts of the sentence. Consequently, RNNs display the missing-VP effect in English (Christiansen & MacDonald, 2009; Engelmann & Vasishth, 2009; Frank et al., 2016).

This view was recently formalized by Futrell et al., (2020) as ‘lossy-context surprisal theory’. A word whose occurrence is less expected has higher surprisal, resulting in longer reading time (Hale, 2001; Levy, 2008). Veridical knowledge of the structure of English (or Dutch, for that matter) should lead to expectations for three VPs after the three NPs of a double-embedded sentence. However, if the memory representation of the sentence is incomplete or incorrect (i.e., because of working-memory limitations) it is possible that the third VP in fact receives high surprisal, that is, longer RT is predicted in the V3 region of grammatical than ungrammatical items, which is exactly what we found in regression-path RTs.

Our first-pass RT results, however, may contradict such a surprisal-based account. These RTs were often longer in the ungrammatical than in the grammatical condition. Specifically, this was the case in the V3 region of semantically biased sentences and post-V region of semantically neutral sentences. This is exactly the pattern one would expect for readers who do not suffer from the grammaticality illusion: Semantically biased ungrammatical sentences are semantically anomalous in the V3 region, whereas in semantically neutral sentences the absence of a verb phrase is not apparent until after the final verb. If first-pass RTs reflect early-stage, expectation-based processes quantified by surprisal, these data are inconsistent with lossy-context surprisal theory (and its implementation in RNNs). Frank and Bod (2011) report stronger surprisal effects on first-pass RT than on regression-path RT, and the best evidence for surprisal as a valid measure of cognitive load during reading comes from first-pass RTs (Goodkind & Bicknell, 2018; Smith & Levy, 2013). Hence, it does appear that surprisal quantifies the cognitive processes measured in first-pass RTs, which is exactly where we (as well as Vasishth et al., 2010) failed to find the missing-VP effect.

### Shallow syntactic processing

According to the shallow structure hypothesis (Clahsen & Felser, 2006a, b), L1 and L2 sentence processing differ in that non-natives rely more on semantic information, in particular for sentences with very complex syntactic structure. Although it is not immediately obvious what this hypothesis predicts for our stimuli, stronger reliance on semantic cues would presumably make it easier to detect something is missing from the ungrammatical sentences, in particular for the semantically biased sentences because these provide semantic cues. Hence, for these sentences, our L1 Dutch participants would be less sensitive to the grammaticality illusion than the L1 English participants. However, our results revealed no three-way interaction between Grammaticality, Semantics, and L1. Proficiency effects within the L1 Dutch group are even in the opposite direction of what the shallow structure hypothesis (arguably) predicts: Lower proficiency resulted in stronger missing-VP effects on semantically biased sentences but not on semantically neutral sentences. If anything, this suggests that lower-proficiency L2 readers rely less on the semantic cues provided by the semantically biased sentences.

This does not mean that our participants always engage in deep syntactic processing. In fact, many participants make close to 20% errors on comprehension questions which suggests they guess the answer in almost 40% of the cases. This could be a consequence of shallow, ‘good enough’ processing (Ferreira & Patson, 2007) but, crucially, it does not differ between native and non-native readers.

## Conclusions

We conducted an eye-tracking reading study to answer the question why L1 Dutch speakers, who do not show the missing-VP effect in Dutch, do show this effect in L2 English: Is this because their knowledge of English is similar to that of native English readers (the high-exposure account) or because L2 reading is more taxing on their working memory (the low-proficiency account)? Our results failed to provide strong and consistent evidence for (or against) either of these explanations, although they do suggest that both high exposure to English and limited proficiency contribute to the presence of the missing-VP effect. This is in line with recent proposals that the effect is caused by an interaction between working-memory capacity constraints and expectations based on statistical word-order patterns.

## Experimental stimuli

Tables 8 and 9 list the semantically neutral and biased target sentences, respectively.

**Table 8:** Grammatically correct, semantically neutral experimental sentences

1	The carpenter who the craftsman who the peasant carried a long | way *hurt on | purpose* supervised the apprentice in the garden.
2	The mother who the daughter who the sister found within | minutes *frightened with | a mask* greeted the grandmother on the tricycle.
3	The worker who the tenant who the foreman looked | for today *injured | with a knife* questioned the shepherd in the office.
4	The trader who the businessman who the professor hired for a certain | period *confused | with questions* annoyed the investor in the morning.
5	The painter who the musician who the father missed by ten | minutes *sheltered in | the attic* cooked for the artist in the kitchen.
6	The saxophonist who the trumpeter who the conductor brought | along *distracted | with flowers* thanked the violinist in his speech.
7	The pharmacist who the optician who the stranger saw from | a distance *troubled with | some remarks* questioned the customer at the counter.
8	The cleaner who the janitor who the doctor recognized by his | uniform *sought | after lunch* surprised the patient in the hallway.
9	The dancer who the singer who the bystander admired with | jealousy *met at | the party* tipped the doorman at the door.
10	The artist who the sportsman who the guard shouted | at yesterday *annoyed | with boring stories* instructed the newscaster in the studio.
11	The son who the father who the teacher saw for the first | time *disturbed by | barging in* visited the grandfather in the nursing home.
12	The defence who the prosecutor who the spy looked at for several | minutes *surprised | by crying* convinced the judge in the courtroom.

Text in italics (presented in normal font in the experiment) was removed to create ungrammatical versions. The first and second vertical line display the location of the line break in the ungrammatical and grammatical condition, respectively. Underlines denote the V3 and post-V regions of interest. Underlines and vertical lines were not visible to participants

**Table 9:** Grammatically correct, semantically biased experimental sentences

1	The book that the student who the new catalog had confused | a great deal *was studying | in the library* was missing an important page.
2	The lullaby that the singer who the record label had signed to a big | contract *was singing | yesterday* was written sixty years ago.
3	The game that the child who the lawnmower had startled in | the yard *was playing in | the morning* lasted for several hours.
4	The crime that the gangster who the story had profiled | thoroughly *had planned | for weeks* was solved in the middle of the night.
5	The picture that the student who the school had expelled for | cheating *was hurriedly | copying* was printed in a popular magazine.
6	The trophy that the athlete who the restaurant had hired as a | spokesman *had won at the | championship* was stolen the day after.
7	The apartment that the maid who the service had sent | over *was cleaning every | week* was decorated with beautiful flowers.
8	The shirt that the seamstress who the officer had investigated | last week *was carefully | mending* needed to be washed at thirty degrees.
9	The lecture that the professor who the newspaper had interviewed in | detail *was teaching | poorly* was attended by twenty students.
10	The novel that the horror author who the publisher had fired | recently *had typed | quickly* was banned by the local library.
11	The prayer that the monk who the religious man had persecuted | fiercly *was chanting | every day* was echoing in the empty church.
12	The play that the actor who the company had underpaid | repeatedly *was performing last | month* was extremely well written by a famous author.

Text in italics (presented in normal font in the experiment) was removed to create ungrammatical versions. The first and second vertical line display the location of the line break in the ungrammatical and grammatical condition, respectively. Underlines denote the V3 and post-V regions of interest. Underlines and vertical lines were not visible to participants

## Footnotes

1 After collecting the data, we discovered that one item misspelled ‘fiercely’ as ‘fiercly’. Because the misspelled word cannot be misunderstood and was not in any of the regions of interest (see below) we decided to include this item in the analysis.

2 PCAs were performed over all L1 Dutch participants, that is, including those that were excluded from analysis.

3 One participant claimed using English 140 hours per week, leaving at most 4 hours per night for sleep. Since this is not realistic, the impossibly high estimates were coded as missing data. The PCA algorithm that was used (alternating least squares) can handle missing data.

4 Note that the choice for a distribution is based on the overall appropriateness of the model for the data, and not on the strength of the effects. Hence, this decision will not artificially strengthen the effects.

5 Following Nicenboim and Vasishth (2016), we consider the evidence for an effect to be “strong” if the credible interval does not contain 0, that is, if *P*(*b* > 0) is above .975 or below .025. Furthermore, evidence for a positive- or negative-going effect is called “weak” if *P*(*b* > 0) ∈ [.95,.975] or *P*(*b* > 0) ∈ [.025,.05], respectively; and “very weak” if *P*(*b* > 0) ∈ [.9,.95] or *P*(*b* > 0) ∈ [.05,.1], respectively.

6 https://github.com/aufrank/R-hacks/blob/master/mer-utils.R

7 To compensate for the larger number of exposure/use measure than proficiency measures (13 and 5, respectively), we used a weighted PCA in which exposure/use and proficiency measures received weights of 5/13 and 1, respectively. Validity of the combined score is confirmed by its negative correlations with log-transformed average filler sentence RT (95% CI [−.34, +.00]) and comprehension question error rate (95% CI [−.44,−.12]).
